# Sprint Performance in Arms-Only Front Crawl Swimming Is Strongly Associated With the Power-To-Drag Ratio

**DOI:** 10.3389/fspor.2022.758095

**Published:** 2022-03-01

**Authors:** Sander Schreven, Jeroen B. J. Smeets, Peter J. Beek

**Affiliations:** ^1^Department of Human Movement Sciences, Faculty of Behavioural and Movement Sciences, Vrije Universiteit Amsterdam, Amsterdam Movement Sciences, Amsterdam, Netherlands; ^2^InnoSportLab De Tongelreep, Eindhoven, Netherlands

**Keywords:** 3d hand kinematics, swimming technique, power, anthropometrics, front crawl, MAD system

## Abstract

To date, optimal propulsion in swimming has been studied predominantly using physical or computational models of the arm and seldom during real-life swimming. In the present study we examined the contributions of selected power, technique and anthropometric measures on sprint performance during arms-only front crawl swimming. To this end, 25 male adult competitive swimmers, equipped with markers on their arms and hands, performed four 25-m sprint trials, which were recorded on video. For the fastest trial of each swimmer, we determined the average swim speed as well as two technique variables: the average stroke width and average horizontal acceleration. Each participant also swam 10–12 trials over a custom-made system for measuring active drag, the MAD system. Since the propelling efficiency is 100% while swimming over the MAD system, the power output of the swimmer is fully used to overcome the drag acting on the body. The resulting speed thus represents the ratio between power output and drag. We included this power-to-drag ratio, the power output and the drag coefficient of the fastest trial on the MAD system in the analysis. Finally, the body height and hand surface area of each swimmer were determined as anthropometric variables. A model selection procedure was conducted to predict the swim speed from the two technique variables, three power variables and the two anthropometric variables. The ratio between power output and the drag was the only significant predictor of the maximal swimming speed (*v* = 0.86·*power/drag*). The variations in this ratio explained 65% of the variance in swimming performance. This indicates that sprint performance in arms-only front crawl swimming is strongly associated with the power-to-drag ratio and not with the isolated power variables and the anthropometric and technique variables selected in the present study.

## Introduction

The overarching aim of competitive swimming is to transverse a given race distance as fast as possible. Swim coaches are therefore constantly looking for ways to improve the swim speed and thus the race performance of their swimmers, as indeed the swimmers do themselves. Two main domains that coaches work on with their swimmers are the mechanical power that can be delivered by the swimmer and the swimming technique employed to convert that power into speed. It is generally understood and experienced that both domains can be altered by training. A third domain that is important for swimming performance concerns the swimmer's anthropometric properties. After maturation the swimmer's anthropometrics are fixed and cannot (or only marginally) be adjusted by training. These three domains have all received attention in studies aimed at identifying relevant performance related variables in speed swimming. In the following sections important findings within each domain are highlighted. An a priori selection of potentially relevant variables from each domain was made based on those findings.

The power balance is a commonly used approach to gain insight into how power, drag and swimming technique affect swimming speed. It posits that the total mechanical power produced by the swimmer (*P*_*o*_) is equal to the power to overcome drag (*P*_*d*_) and the power expended in pushing away masses of water (*P*_*k*_). Unlike the ground surface in running, water is a non-stationary medium that is brought into motion during the push-off (van Ingen Schenau and Cavanagh, 1990; Rodríguez and Mader, 2011). The theoretical relationship between swim speed (*v*), power output (*P*_*o*_), propelling efficiency (*e*_*p*_) and drag (represented by the drag coefficient *K*) shows how power output and propelling efficiency both contribute to swimming speed (Toussaint and Truijens, 2006; Rodríguez and Mader, 2011):


(1) $$\begin{array}{l} v=\sqrt[{3}]{\frac{P_{o}\cdot e_{p}}{K}} \end{array}$$

The power balance for swimming illustrates that swimmers have two main options to swim faster, namely to increase their overall power and to decrease the power losses associated with overcoming drag and bringing water into motion. The first option may be realized through strength training, which makes swimmers stronger and capable of generating greater power, while the second option may be realized by optimizing swimming technique, resulting in a higher propelling efficiency.

In the power domain, numerous studies have determined power output on land and some in water. Several studies reported a significant relationship between swimming performance and dry land power tests in which power output was determined with an upper-body ergometer (e.g., Hawley and Williams, 1991; Hawley et al., 1992; Zamparo et al., 2014), swim bench (arms only: e.g., Sharp et al., 1982, whole-body: e.g., Gatta et al., 2017) or during strength exercises (e.g., Pérez-Olea et al., 2018), although some of these studies also included variables and/or considerations related to the technique and anthropometric domain. Significant correlations were also found between power tests in the pool and swimming performance. In the water, power was determined with semi-tethered (e.g., Costill et al., 1983; Dominguez-Castells et al., 2013) swimming tests and by using the MAD system (Toussaint and Vervoorn, 1990). However, the high correlation coefficients found in some of these studies should be regarded with caution since the participants in these studies were heterogeneous in terms of age and gender (Morouço et al., 2012). Nevertheless, the general conclusion that can be drawn from the literature is that the power output of the swimmer in relation to drag is an important determinant of swimming performance.

In the technique domain, the trajectory, orientation, speed and acceleration of the hand are aspects of the swimming technique that have been studied extensively, particularly in front crawl swimming, while other aspects such as leg, trunk and head movements have received considerably less attention. In an encompassing literature review, van Houwelingen et al. (2017) summarized the current state of knowledge regarding the hydrodynamic aspects of hand and arm movements in front crawl swimming. Since the influential work of Counsilman (1971), there has been considerable debate in the literature whether or not the hand trajectory in the front crawl stroke should contain lateral (sculling) movements. van Houwelingen et al. (2017) concluded from the literature that excessive sculling movements generally lead to lower propulsive forces than a (roughly) straight underwater stroke and should therefore be avoided. With respect to optimal hand orientation no firm conclusions could be drawn, since the results reported on this variable were too inconsistent. van Houwelingen et al. (2017) further concluded that accelerating the hand leads to a higher propulsive force compared to a stroke performed at constant speed, implying that a high acceleration would be desirable for effective propulsion.

In the anthropometric domain, body height, hand surface area and arm span have been associated with swimming performance. In several studies (Klentrou and Montpetit, 1991; Geladas et al., 2005; Lätt et al., 2010) a significant correlation between body height and swimming performance was found in young swimmers. Moura et al. (2014) found that body height was a significant predictor of the propulsive arm force in young swimmers, even after having controlled for maturation stage. Two potential mechanisms are described in the literature through which body height could be positively related to swimming speed. First, it has been suggested that an increased body height could reduce the wave drag acting on the body (Toussaint et al., 1990, 2000; Toussaint and Beek, 1992). Second, taller swimmers were found to have larger arm spans, which in turn were found to be associated with increased stroke length and swimming performance (Grimston and Hay, 1986; Mazzilli, 2019).

The beneficial effect of a large hand surface area on swimming performance can be understood best from the equations that describe the forces acting on the hand and arm during the stroke. These forces are typically described in a component parallel to the line of hand motion, the so-called drag forces, and a component perpendicular to the line of hand motion, the so-called lift forces. The drag and lift forces acting on the hand can be derived from the following equation:


(2) $$\begin{array}{l} F_{D,L}=\;\frac{1}{2}\rho Av_{hand}^{2}C_{D,L} \end{array}$$

where ρ is the water density, *A* is the hand surface as projected on a plane perpendicular to the mean flow (for the drag force), *v*_*hand*_ is the hand speed, and C_*D, L*_ is the drag/lift coefficient (Toussaint and Beek, 1992; van Houwelingen et al., 2017). Since the projected hand surface area *A* is directly related to the forces acting on the hand, a large hand surface area seems an important anthropometric asset for competitive swimmers besides body height.

The cited findings in the three domains suggest that factors related to power, propulsion technique and anthropometrics all contribute to swimming performance. One point of concern is that for the most part, the conclusions drawn by van Houwelingen et al. (2017) about optimal swimming technique are either based on studies in which physical arm models were equipped with actuators and/or sensors or studies in which a computational fluid dynamics model was simulated, while only few studies investigated optimal swimming technique during actual swimming. Another point of concern is that most studies only looked at the effect of one of the domains of swimming performance distinguished here, instead of adopting an integral approach covering variables from all three domains. In one of the few studies that looked at more than one domain, Klentrou and Montpetit (1991) found that height, arm span, maximal stroke rate and power, measured using a tethered swim, were predictors of 100 m performance in 25 male age-group swimmers. The model containing height and arm span explained 56% of the variance. After adding the measured power to the model, the explained variance increased by 10% to a total of 66%. The maximal stroke rate added another 5% of the explained variance. Whereas the participants in the study of Klentrou and Montpetit (1991) were age-group swimmers, Lätt et al. (2010) concluded that technique factors (stroke rate and stroke index) explained 90.3% of the variance in 100 m sprint performance in adolescent male swimmers. Anthropometric factors explained 45.8% of the variance. The participants in the studies of both Klentrou and Montpetit (1991) and Lätt et al. (2010) were youth swimmers. For adult swimmers the contribution of each domain might be different. Moreover, in none of the studies in question factors from all three domains—i.e., power generation, propulsion technique and anthropometrics—were included and compared.

In the present study we adopted an integral approach aimed at determining and comparing the contributions of selected power, technique and anthropometric measures on sprint performance during arms-only front crawl swimming in adult, male competitive swimmers. Based on the literature, we expected that variables from each of the three domains would contribute to swimming performance.

## Materials and Methods

Twenty-five healthy, male adult competitive swimmers [age: 22 ± 5 years, body weight: 77.6 ± 9.2 kg, body height: 184.8 ± 6.4 cm; all measures mean ± standard deviation (SD)] participated in the study. For each participant, the highest FINA score (based on the FINA 2018 points table; Kaufmann, 2018) during competition within the period between 90 days before and 90 days after the measurement day was obtained from www.swimrankings.net. The participants scored 593 ± 108 FINA points within this period. Their average personal best time (also obtained from swimrankings.net) on the 50 meter and 100 meter freestyle (long course) were, respectively, 25.8 ± 1.5 s and 56.1 ± 2.5 s. The participants volunteered to partake in the study following an informal recruitment procedure via their swimming club or coach, and provided informed consent prior to the start of the study. Only male swimmers, 18 years or older, with a personal best below 60 s on the 100 m freestyle (long course) were included in the study. The protocol for the study was approved by the local ethics committee of the Faculty of Behavioural and Movement Sciences of the Vrije Universiteit Amsterdam (VCWE, VCWE-2018-054). The protocol consisted of three parts: (1) anthropometric measurement, (2) measurement of hand kinematics during arms-only front crawl swimming, and (3) measurements of power output and drag.

### Anthropometric Measurements

Upon arrival at the InnoSportLab De Tongelreep at Eindhoven, where the study was conducted, participants were informed about the general aim and the experimental procedures of the study. Subsequently, a series of anthropometric measures were taken, including body height and hand surface area. The hand surface was measured using the available equipment in the testing environment. This was done as follows. First, one of the experimenters marked the location of the ulnar and radial styloid on the skin of the right arm with a pencil. Next, the participant placed his right hand flat on a vertical surface with fingers spread. Perpendicular to the surface, a camera (Sony NEX-VG20E) was positioned to take a picture of the hand. A sheet of A4 paper was placed on the same surface close to the participant's hand. The resulting image was postprocessed in ImageJ and rescaled using the known distance of the long side of the A4 paper. Finally, the hand surface area was determined by tracing the hand until the skin marks of the ulnar and radial styloid.

### Measurement of Hand Kinematics During Arms-Only Front Crawl Swimming

After the participants had been prepared for measurement, they swam for 15 min to warm up and familiarize themselves with swimming with clusters of LED markers attached to the ventral and dorsal side of both forearms and markers placed on the tip of the middle fingers. Immediately thereafter they performed four trials in front crawl starting from the middle of the 50-m long pool (i.e., at 25 meters) toward the wall. The swimming movements were recorded within a calibrated volume of 2 × 1 × 1 m (i.e., 2 m long in the swimming direction). The participants were instructed to swim as fast as possible in each trial. Their legs were supported by a pull-buoy and they were instructed not to use the leg kick. Since breathing has an effect on the stroke kinematics, they were also instructed not to breathe around the calibrated volume.

In the pool, cameras (scA1400-30gc, Basler AG, Ahrensburg, Germany, 50 fps) in the sidewall of the pool positioned at, respectively, 15 and 5 m from the start edge of the pool were used to determine the average swimming speed (*v*_*trial*_) in this segment, while six cameras (avA1900-50gc, Basler AG, Ahrensburg, Germany, 50 fps) in underwater housings placed at the bottom of the pool were used to capture the movement of the right arm. The intrinsic parameters of the cameras at the bottom of the pool were determined with the Camera Calibration Toolbox in Matlab (Bouguet, 2008) using a checkerboard, while the Direct Linear Transformation (DLT) parameters were calculated based on a 2 × 1 × 1 m calibration frame containing 60 control points. These parameters were combined with the tracked marker positions to reconstruct the real-world coordinates in 3D. The position of the marker on the middle finger was tracked frame-by-frame by the experimenter using custom-made software. If the experimenter could not judge the position of a marker, the missing data were filled by linear interpolation. These raw data were filtered using a second order low-pass Butterworth filter. A cut-off frequency of 10 Hz was used to filter the coordinates of the marker on the tip of the middle finger. This cut-off frequency was chosen based on the results of a previous study showing that the optimal dynamic precision of a marker cluster modeled on the forearm was smallest with a cut-off frequency of 10 Hz (Schreven et al., 2015). The tracking procedure was only conducted on one stroke of the right arm in the fastest trial of each swimmer, resulting in 25 observations. Since the aim of the present study was to predict maximal swim speed from power, technique and anthropometric variables, only the fastest trial of each swimmer was included in the analysis.

Based on the processed real-world coordinates, two technique variables were calculated: the stroke width (standard deviation of the lateral position of the tip of the middle finger) and *a*_*hand, hor*_ (mean absolute horizontal acceleration of the tip of the middle finger). Ideally, the technique variables would be calculated over the full backward part of the stroke from the moment that the tip of the middle finger starts moving backwards (*t*_0_, *t* = 0%) until the last frame in which the tip of the middle finger was visible in the underwater recordings (*t*_100_, *t* = 100%). However, since the markers were not visible in each trial from *t*_0_ to *t*_100_ the part of the stroke in which data was available for all participants had to be determined. The marker at the tip of the middle finger was visible for all participants from *t* = 0 to *t* = 90% (*t*_90_) and therefore the technique variables stroke width and *a*_*hand, hor*_ were calculated between *t*_0_ and *t*_90_.

### Measurement of Power Output and Drag

To obtain variables describing drag and power output, a system dedicated to this purpose was used, the so-called measuring active drag or MAD system (Hollander et al., 1986). The MAD system is one of the established methods to measure active drag (for an overview of all established methods for this purpose see Toussaint et al., 2000; Wilson and Thorp, 2003). The MAD system consists of a 23-meter long rod with 17 push-off pads attached to it. The rod is positioned 0.8 meters below the water surface. The distance between the pads is 1.35 m and the top edge of the push-off pads is positioned 0.56 m below the water surface. The dimensions of each push-off pad are 25.5 × 16.5 cm. The rod is attached to a waterproof force transducer (BSP-603, Vishay Precision Group, Malvern, Pennsylvania, USA) connected to the wall. The force signal is digitized with an A/D converter (NI 9237 and cDAQ-9171, National Instruments, Austin, Texas, USA) at a sampling rate of 100 Hz. The participant swims over the system by pushing off against the fixed push-off pads. The propelling efficiency is 100% while swimming over the MAD system, because the swimmer pushes off against a fixed surface and no power is lost by pushing away masses of water. The speed reached by the participant is therefore determined by the power-to-drag ratio and thus represents a direct measure for this ratio.

Each participant swam five trials of 23 m on the MAD system to become familiar with swimming over the system. Next, the participant swam 10–12 trials over the system during which data were recorded, starting at a speed around 1.2 m/s and incrementally increasing the speed each trial by ~0.1 m/s until the maximal speed of the participant in question was reached. Next, one extra attempt was made at maximal intensity. The breaks between adjacent trials lasted ~3 min. During all trials, participants swam over the system with a pull buoy between their thighs to provide support to the body without kicking their legs. They were also instructed not to breathe while swimming over the system. Using a custom made Matlab script, the force data were filtered using a 2nd order low-pass Butterworth filter with a cut-off frequency of 10 Hz. The average swim speed in each trial on the MAD system was determined by manually selecting the time interval between the onset of the push-off against the second push-off pad and the onset of the push-off against the last (17th) push-off pad. During the same interval the average push-off force was determined. For each participant the maximal power-to-drag ratio (as determined by the maximal average speed achieved on the MAD system) was used in the statistical analysis. We will refer to this as “*power/drag”*. Furthermore, the power output and drag coefficient were determined for the trial in which the maximal average speed was achieved. The power output was calculated by multiplying the average push-off force by the average speed. The drag coefficient was determined by dividing the average push-off force by the average speed squared. Since we obtained three power variables (*power/drag*, power output, and drag coefficient) from two measurements (average speed and average force in the fastest trial on the MAD system), we expected redundancy between those variables. Therefore, we checked in the statistical analysis for collinearity between these (and all other) independent variables to reduce the redundancy between the power variables.

### Statistical Analysis

The statistical analysis was performed in R (R Core Team, 2020) using RStudio 1.3.1056 (RStudio, Boston, Massachusetts, USA). The following R packages were used: nlme (Pinheiro et al., 2020), readxl (Wickham and Bryan, 2019) and regclass (Petrie, 2020). The aim of the analysis was to determine the optimal model to predict *v*_*trial*_ (dependent variable) from the following seven independent variables: body height, hand surface area, *power/drag*, power output, drag coefficient, stroke width and *a*_*hand, hor*_. The technique variables and swim speed from the fastest trial out of the four trials were included in the dataset, resulting in a total of 25 observations. First, boxplots and histograms were made for all independent variables to detect outliers. Collinearity was assessed by calculating Pearson's correlation coefficient between all independent variables. In case two variables had a Pearson's correlation coefficient above 0.7, one independent variable was selected.

In line with the procedure described by Zuur et al. (2009), the following three steps were taken to construct the optimal model taking into account both fixed effects and the residual variance structure using the generalized least squares technique. First, the optimal residual variance structure was determined. All independent variables were entered as fixed terms in the model. Models with different residual variance structures were compared using the Akaike information criterion (AIC). A total of 14 residual variance structures were compared: seven models with a fixed variance with each of the independent variables as variance covariate and seven models with a “power of the covariate” variance structure with each of the independent variables as variance covariate; the residual variance structure that resulted in the smallest AIC was selected as the optimal residual variance structure. The various residual variance structures were also compared to a standard linear model. In this first step the model parameters were estimated using the Restricted Maximum Likelihood approach (REML). Second, using the optimal residual variance structure selected in the previous step, a step down procedure was followed to find the optimal fixed structure starting by entering all independent variables as fixed terms in the model. In each round of the parameter removal procedure all fixed terms were dropped one by one and using the likelihood ratio test each of the models in which one of the fixed terms was dropped was compared to the full model from the start of the elimination round. In case any of the fixed terms was not significant (*p* > 0.05), the parameter with the highest *p*-value in the likelihood ratio test was removed from the model and a new round of the elimination process was started. This process was repeated until all fixed terms in the model were significant. In this second step the model parameters were estimated using Maximum Likelihood estimation. Third, the results of the model selected in the second step were presented using the values obtained by REML estimation.

## Results

**Figure 1:**
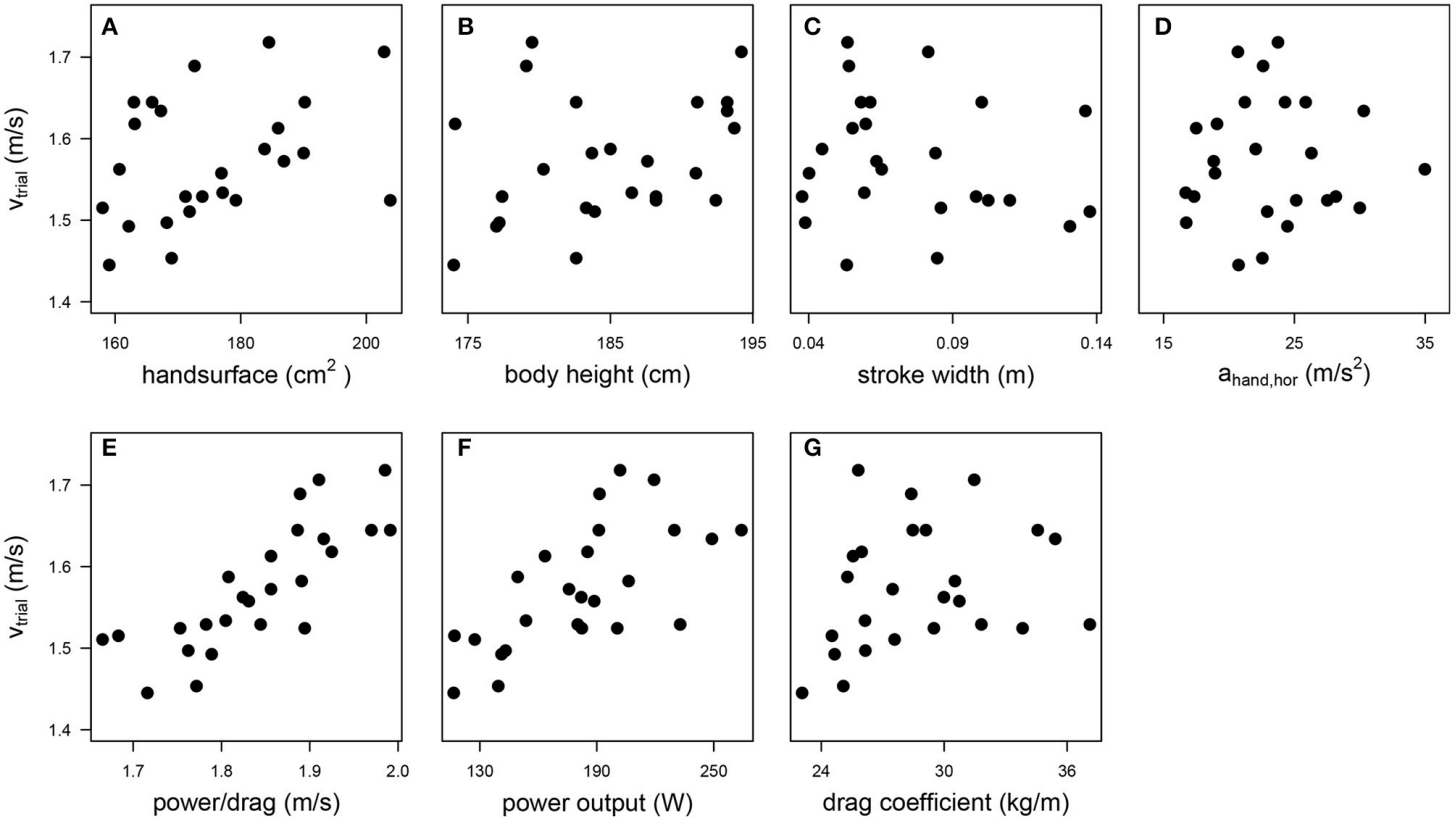
Scatter plots of the maximal swim speed (*v*_*trial*_) as a function of the 7 independent variables: hand surface **(A)**, body height **(B)**, stroke width **(C)**, the mean horizontal hand acceleration (*a*_*hand, hor*_, **D**), power-to-drag ratio (obtained from the speed of the fastest trial on the MAD system, *power/drag*, **E**), power output **(F)**, and drag coefficient **(G)**.

**Figure 2:**
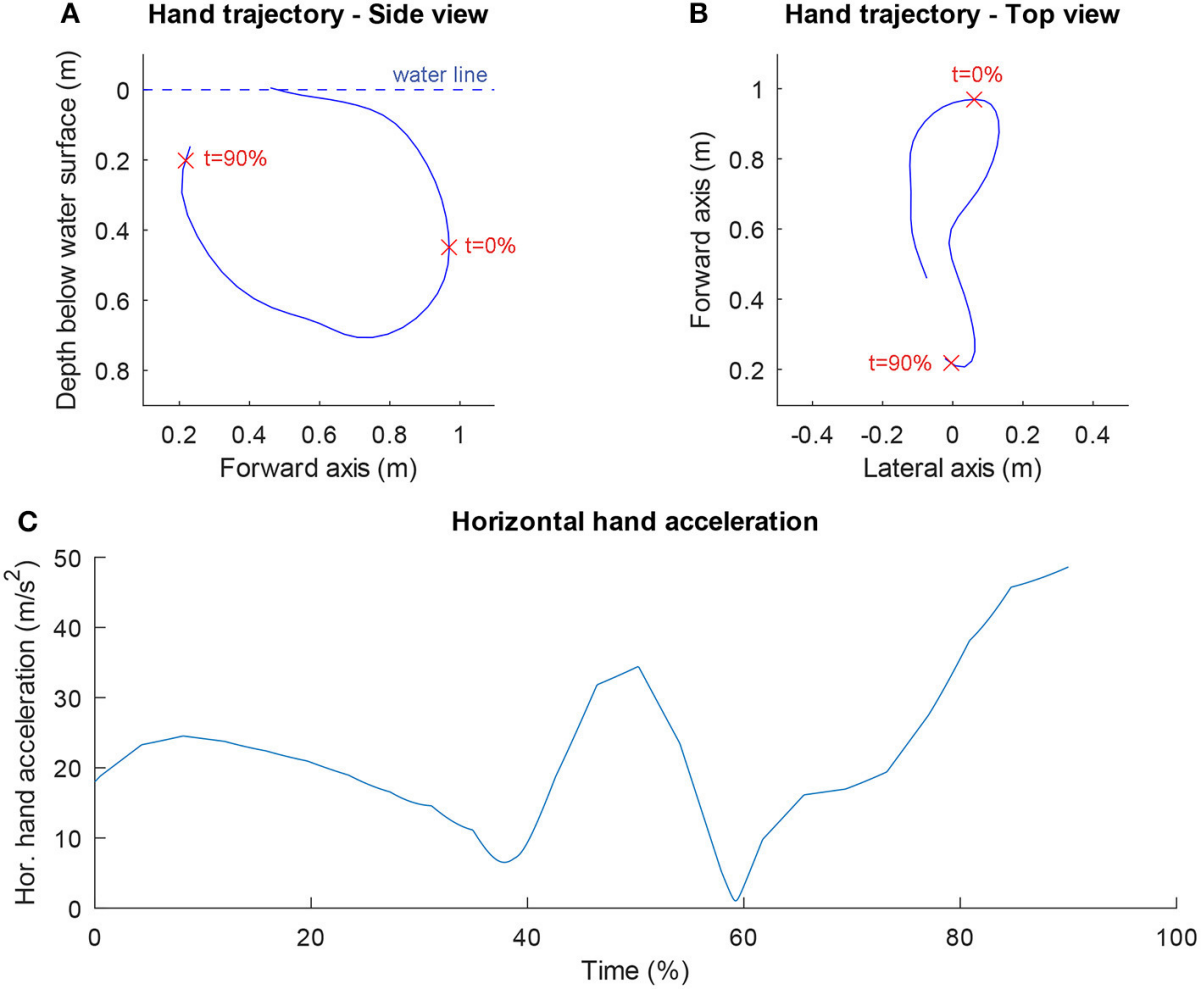
Example of hand trajectory. The side view **(A)**, top view **(B)**, and of one of the swimmers is presented together with the horizontal hand acceleration **(C)**. The stroke width was 0.045 m and the mean horizontal hand acceleration was 22.0 m/s^2^ in this trial. The red crosses in **(A,B)** indicate the limits of the part of the stroke that was analyzed (see Methods section).

Table 1 shows an overview of the results for the dependent and independent variables. The swimmers could indeed swim fast (1.57 ± 0.08 m/s). As expected, they swam even faster on the MAD system (1.84 ± 0.09 m/s) because in this environment no power is lost by bringing water into motion. The variation in the anthropometric variables was smaller (coefficient of variation < 0.1) than in the technique variables. As can be seen in Figure 1, which shows the scatter plots of the maximal swim speed as a function of all independent variables, there were no outliers for any of the variables. A high correlation coefficient (*r* > 0.70) was found between *power/drag* and power output (*r* = 0.83) and between power output and the drag coefficient (*r* = 0.80), indicating collinearity between the variables in question. Power output was therefore excluded as independent variable, because the correlation coefficient with *v*_*trial*_ was higher for *power/drag* (*r* = 0.81) than for power output (*r* = 0.66). To indicate how a hand trajectory leads to values for the two technique variables, the hand trajectory in side-view (top, left panel) and top-view (top, right panel) and the horizontal hand acceleration (bottom panel) are shown for one of the swimmers in Figure 2.

**Table 1:** Overview of the values of the dependent and independent variables.

**Variable**	**Mean ±SD**	**95% Confidence interval**
*v*_*trial*_ (m/s)	1.57 ± 0.08	(1.54, 1.60)
body height (cm)	184.8 ± 6.4	(182.1, 187.4)
hand surface area (cm^2^)	175.5 ± 12.9	(170.2, 180.8)
*power/drag* (m/s)	1.84 ± 0.09	(1.80, 1.88)
power output (W)	181 ± 40	(165, 198)
drag coefficient (kg/m)	28.7 ± 3.7	(27.2, 30.3)
*a*_*hand, hor*_ (m/s^2^)	23.1 ± 4.7	(21.2, 25.1)
stroke width (m)	0.076 ± 0.030	(0.063, 0.088)

*Values are given over the 25 observations*.

In search for the optimal residual variance structure, the model with a fixed variance structure with stroke width as the variance covariate had the lowest AIC value (AIC = −20.7) and was therefore preferred over the standard linear model (AIC = −17.8), the model with a power to the covariate structure with stroke width as variance covariate (AIC = −20.2) and the models in which the other independent variables were used as variance covariates (AIC > −18.3). In search for the optimal fixed effects, the non-significant independent variables were eliminated in the following order: intercept (*L* = 0.03, *p* = 0.86), hand surface area (*L* = 0.29, *p* = 0.59), drag coefficient (*L* < 0.80, *p* = 0.37), body height (*L* = 1.44, *p* = 0.23), *a*_*hand, hor*_ (*L* = 1.18, *p* = 0.28) and stroke width (*L* = 0.38, *p* = 0.54). Only *power/drag* was a significant predictor of the maximal sprint speed [mean: 0.856, 95% confidence interval (0.847, 0.865)] resulting in the following model:


(3) $$\begin{array}{l} v_{trial}=0.856\cdot \;power/drag \end{array}$$

**Figure 3:**
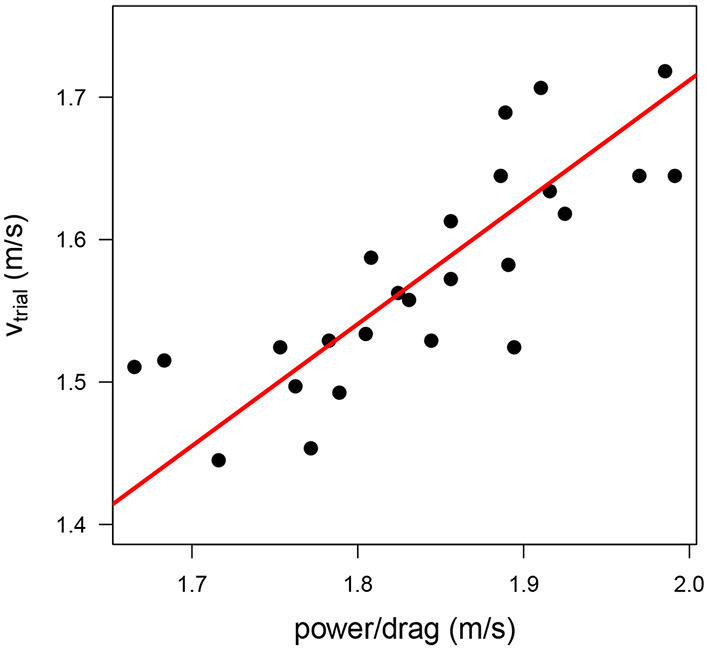
Scatter plot of the maximal swim speed (*v*_*trial*_) as a function of the power-to-drag ratio (obtained from the speed of the fastest trial on the MAD system, *power/drag*). The red line indicates the regression line based on the optimal model (*v*_*trial*_ = 0.856 · *power/drag*).

The scatter plot of *power/drag* vs. *v*_*trial*_ in Figure 3 shows the strong relationship between both variables. A strong positive correlation (*R*^2^ = 0.65) between both variables indicated that the variations in the maximal power-to-drag ratio explained 65% of the variance in swimming performance.

## Discussion

The aim of the present study was to determine which variables from the power, technique, and anthropometric domain contribute significantly to the prediction of maximal sprint speed during arms-only front crawl swimming. We expected variables from all three domains to be significant predictors. However, the results showed that the maximal power-to-drag ratio, determined by the maximal speed swum on the MAD system, was the only significant predictor in the model. Unexpectedly, given previous findings reported in the literature, the technique and anthropometric variables selected in this study were excluded from the final model. The resulting model parameter indicates that a 1 m/s higher maximal power-to-drag ratio was related to a 0.86 m/s higher maximal sprint speed during the swimming trials. The variations in the maximal power-to-drag ratio explained 65% of the variance in the swimming performance.

The present study has several limitations that need to be discussed. The technique variables were obtained from only one stroke of the right arm, while competitive swimmers make many more strokes per lap. It may be questioned whether the technique variables obtained during that single stroke provide a valid representation of the technique of the swimmer in question as there will be a degree of variability in the arm movements and more technique variables might contribute to swimming performance than considered in the present study. For example, we intended to include hand orientation to the technique variables because it follows from Equation (2) that the hand area projected on the plane of the flow is important for the propulsion generated by the arm. Several studies (e.g., Berger et al., 1995; Bixler and Riewald, 2002; Sato and Hino, 2003) on numerical and physical arm models found that the drag and lift coefficients determined in a steady state flow varied with the angle of attack, although the results reported on this variable were too inconsistent to draw firm conclusions (van Houwelingen et al., 2017). We tried to determine this variable by means of marker clusters attached to the forearm. However, this method proved unreliable, as orientations obtained from a marker cluster placed on the dorsal side of the forearm deviated substantially from the orientation obtained from a cluster placed on the ventral side. This could have been caused by skin movement artifacts but this is uncertain; more research is needed to resolve this issue and to determine where these (rigid) marker clusters should be placed on the swimmer's body. Since we were unable to determine hand orientation reliably, we could not test whether it accounted for some of the variance in swim speed. Furthermore, the experimental task was restricted to arms-only front crawl swimming: the legs were not used for propulsion and supported by a pull buoy in all sprints, which affects the swimmer's body position in the water and leads to slower sprint speeds. Despite these restrictions, *v*_*trial*_ was significantly correlated with the personal best times on the 50 meter freestyle (*r* = −0.69, *p* < 0.01) and 100 meter freestyle (*r* = −0.52, *p* < 0.01).

Not only did we exclude the contribution of the legs, we also ignored the inter-arm coordination (which can be quantified with the index of coordination; Chollet et al., 2000), and the coordination between the arms and legs. The variables selected in this study represent a subset of all possible variables that could be included from the three domains of interest. Patently, the hydrodynamics during actual swimming is much more complex than is covered by our current quantification of technique in terms of mean stroke width and mean horizontal hand acceleration. It is very likely that an interaction occurs between hand path, speed, acceleration, and orientation, which might be oversimplified with the selected technique variables.

Another limitation was the relatively small number of participants in this study. Larger sample sizes would lead to more robust results and allow independent variables with small contributions to be included in the model. Furthermore, with a larger number of observations more variables from each domain could be included. The a priori selection of variables would then likely include more variables that predict swimming speed. The digitization process to obtain the technique variables remains very time-consuming and precludes the inclusion of many participants in studies involving a detailed analysis of the hand kinematics during actual swimming. Although the participants in the present study were all competitive swimmers, the average personal best times and FINA scores as reported in the Methods section indicate that the majority of the participants were no elite swimmers. The results might well be different for a sample consisting solely of elite swimmers.

A crucial limitation is that to date no gold standard exists to determine power output and drag in swimming. All of the established methods have their limitations and underlying assumptions and cross-validations between various pairs of these methods have shown limited agreement (Toussaint et al., 2004; Formosa et al., 2012; Mason et al., 2013). In the present study, the MAD system was used to determine power output and drag. One of the limitations of the MAD system is that the push-off pads on the MAD system were placed at a fixed inter-pad distance of 1.35 m. Although it was found that different inter-pad distances did not affect the measured drag (Schreven et al., 2013), it has not been studied to date whether the maximal speed achieved on the MAD system varies with inter-pad distance. Therefore, it cannot be excluded that by using a different inter-pad distance, the correlation with swimming speed would have been different. Given this discussion on the validity of the various methods to measure power and drag, it is an important open question to what extent the association between power-to-drag ratio and sprint performance depends on the method used to determine power output and drag.

In contrast to the many studies that reported important contributions of the technique and anthropometric domains to swim speed, the variables from these domains did not add significantly to the prediction of sprint speed in our study. One possible explanation for this lack of effect might be that we only selected a limited set of variables from the technique and anthropometric domain, as discussed above. However, as we chose the variables that, based on the literature, were most likely associated with swimming performance, we deem this explanation less likely. An alternative explanation might be that these variables did not contribute significantly to the variation in sprint speed between participants in the current group, as the group consisted of well-trained competitive swimmers that have passed various selection stages. Indeed, the participants in the studies that reported a significant correlation between body height and swim performance were all youth swimmers (Klentrou and Montpetit, 1991; Geladas et al., 2005; Lätt et al., 2010).

This leaves us with the general conclusion that a substantial portion (i.e., 65%) of the variance in maximal swim speed is explained by the maximal speed on the MAD system. Although we introduced the maximum speed on the MAD system as a power related variable, it represents in fact the power-to-drag ratio, as explained in the Methods section. As the drag is determined by anthropometric factors, this variable also reflects some anthropometric characteristics of the participant. Moreover, the MAD system forces the participant to use a certain stroke length, which might differ from the participant's preferred stroke length. The maximum speed on the MAD system might therefore also reflect an aspect of the technique domain.

The strong relationship between the maximal power-to-drag ratio and the maximal swim speed indicates that the maximal power-to-drag ratio is strongly associated with sprint performance. It remains to be explored whether a cause-and-effect relationship exists between both variables. The maximal power-to-drag ratio could be the swimming equivalent of power-to-bodyweight ratio that is considered a key performance indicator in for example cycling (Faria et al., 2005), especially when cycling uphill (Antón et al., 2007). The correlation suggests that increasing the power output by strength training might be beneficial for swimming performance, provided that the positive effect of the increase in power output outweighs the potentially negative effect of an increase in frontal area due to muscle hypertrophy. The MAD system allows determining the maximal power-to-drag ratio in a time effective manner. The participants did not have prior experience with the MAD system and were able to complete the protocol for the power measurements within 30 min. This system can therefore be used to evaluate changes in the maximal power-to-drag ratio due to training and might be an expedient way to identify talented swimmers, irrespective of their technique, although it remains to be established whether the maximal power-to-drag ratio determined at a young age predicts swim performance at a later, more senior age.

Future research should aim for a better understanding of the role of power, technique and anthropometrics, as well as the underlying mechanisms. Furthermore, the relative contribution of each of these domains on swim performance should be studied for swimmers of different age, sex and swim level as the (relative) contribution might be different in other populations. Also, since the maximal power-to-drag ratio was found to be an important predictor of swim speed, it would be interesting to investigate whether a causal relationship exists between both variables and if so, which strength training interventions lead to the largest improvement of this ratio and what would be the optimal muscle architecture to maximize this ratio.

## Data Availability Statement

The raw data supporting the conclusions of this article will be made available by the authors, without undue reservation.

## Ethics Statement

The studies involving human participants were reviewed and approved by the Local Ethics Committee of the Faculty of Behavioural and Movement Sciences of the Vrije Universiteit Amsterdam (VCWE, VCWE-2018-054). The patients/participants provided their written informed consent to participate in this study.

## Author Contributions

SS conducted the experiment and collected the data. SS and JS performed the statistical analysis. SS and PB wrote the first draft of the manuscript. All authors contributed to the conception and experimental and statistical design of the study and contributed to subsequent versions of the manuscript, read, and approved the final version.

## Conflict of Interest

The authors declare that the research was conducted in the absence of any commercial or financial relationships that could be construed as a potential conflict of interest.

## Publisher's Note

All claims expressed in this article are solely those of the authors and do not necessarily represent those of their affiliated organizations, or those of the publisher, the editors and the reviewers. Any product that may be evaluated in this article, or claim that may be made by its manufacturer, is not guaranteed or endorsed by the publisher.
